# How parental autonomy support fosters adolescent future orientation: the mediating roles of growth mindset and hope, moderated by peer relationships

**DOI:** 10.3389/fpsyg.2025.1609303

**Published:** 2026-02-09

**Authors:** Xiaohui Li, Asad Ur Rehman Awan, Tianyong Chen

**Affiliations:** 1 State Key Laboratory of Cognitive Science and Mental Health, Institute of Psychology, Chinese Academy of Sciences, Beijing, China; 2 Department of Psychology, University of Chinese Academy of Sciences, Beijing, China

**Keywords:** parental autonomy support, future orientation, growth mindset, hope, peer relationships

## Abstract

Grounded in positive youth development (PYD) perspective and the developmental assets framework (DAF), this study examined a moderated chain mediation model to investigate how perceived parental autonomy support (PAS) was associated with adolescent future orientation (AFO) through the sequential mediators of growth mindset and hope, and how peer relationships moderated these pathways. Data were collected from 604 middle school students in suburban Beijing using validated scales measuring PAS, AFO, growth mindset, hope, and peer relationships. The findings showed that: (1) PAS was significantly associated with more positive AFO. (2) Growth mindset and hope sequentially mediated the relationship between PAS and AFO, indicating that PAS was associated with a growth mindset, which in turn was linked to higher hope, ultimately correlating with a stronger future orientation. (3) Peer relationships moderated the initial link between PAS and growth mindset: the positive effect of PAS on growth mindset was stronger for adolescents reporting higher quality peer relationships. However, peer relationships did not moderate the association between PAS and hope. Conclusions: These findings suggest the mechanisms through which parental autonomy support facilitates adolescents’ future orientation, highlighting the crucial sequential roles of growth mindset and hope. The moderating effect of peer relationships explains the importance of the broader social context in shaping how parental autonomy support influences adolescents’ core beliefs about their abilities. This research contributes to understanding positive youth development and has implications for interventions aimed at fostering adolescents’ future planning and resilience.

## Introduction

Future orientation, conceptualized as a cognitive-motivational process wherein individuals contemplate, plan for, and shape their future (Nurmi, 1990), is particularly crucial for early adolescents’ adaptability and positive development in an increasingly volatile, uncertain, complex, and ambiguous (VUCA) era. Early adolescents (typically aged 12–14), who are beginning to form a coherent sense of self and explore future possibilities, need to develop a positive future orientation to cope with increasingly complex social environments and the growing demands for adaptability and holistic development. Systematic reviews affirm that future orientation is a key promotive factor for positive youth development (e.g., academic achievement and mental health) and a protective buffer in risky environments (Coulter et al., 2022). Empirical evidence demonstrates that positive future orientation assists these youth in managing unpredictable environments and promotes their holistic development. Furthermore, it contributes to effective stress regulation and the reduction of anxiety and depression (Zeng et al., 2022), while concurrently enhancing career resilience and the ability to cope with uncertainty (Khampirat, 2020). Grounded in the positive youth development perspective (Roth and Brooks-Gunn, 2003) and the developmental assets framework (Benson et al., 2003, 2011) that outlines a “facilitative ecosystem” of external and internal assets, this study specifically investigates parental autonomy support as a pivotal external asset. While existing literature indicates that parental autonomy support promotes the internalization of future goals by fulfilling adolescents’ basic psychological needs (Ryan and Deci, 2017), the precise mechanisms through which it influences future orientation are not yet fully understood. As Chang and Zhang (2013) highlighted, clarifying how external and internal assets interact to foster positive development remains a crucial theoretical objective. Therefore, this study examines these assets as an integrated system to elucidate the formative mechanisms underlying early adolescents’ positive future orientation.

### Relationship between parental autonomy support and positive future orientation

Parental autonomy support, functioning as a key initiating hub for external assets within the family system, is conceptualized as a parenting-specific relational behavior that promotes the adolescent’s authentic self-endorsement of actions rather than merely encouraging behavioral independence (Ryan and Deci, 2017). It is operationalized through four key parental practices: (a) acknowledging the child’s perspective and feelings, (b) providing meaningful rationales for expectations, (c) offering developmentally appropriate choices, and (d) minimizing controlling language and pressure (Joussemet et al., 2008). Beyond facilitating relational harmony, parental autonomy support is theorized to fulfill a key developmental function, as it is associated with the development of adolescents’ capacity for constructive goal-setting, mature decision-making, and future-oriented agency (Vasquez et al., 2016). Within cultural contexts characterized by high academic expectations and prevalent parental control (e.g., China; Cheung and Pomerantz, 2011), Parental autonomy support acts as a constructive counterbalance to psychological control, fostering healthy development not by lowering standards but by supporting the child’s volitional engagement and autonomous growth.

The volition-supportive nature of autonomy support is theorized to directly foster adolescents’ future orientation. A meta-analysis consolidating extensive evidence confirms that parental autonomy support is a robust promotive factor for key adolescent outcomes, including academic achievement and psychosocial functioning (Vasquez et al., 2016). This supportive influence is further understood within relational and systemic contexts. Research indicates that adolescent adjustment is linked not only to the level of support provided but also to the congruence between parent and adolescent perceptions of that support (Zheng and Chen, 2025). Moreover, longitudinal studies show that the co-occurring trajectories of autonomy support and other parenting behaviors (e.g., psychological control) jointly shape long-term developmental outcomes (Luo et al., 2025). Converging evidence specifically links autonomy support to enhanced future-oriented capacities, such as proactive planning, goal internalization, and a positive outlook (Ginevra et al., 2015; Ryan and Deci, 2017). While acknowledging that this relationship may be further influenced by a constellation of individual, social, and contextual factors, we hypothesize, based on the foregoing empirical and theoretical foundation, that parental autonomy support will be positively associated with early adolescents’ positive future orientation.

### The mediating effect of growth mindset (as an internal asset)

A growth mindset is an intrinsic belief held by individuals that fundamental human abilities can be developed through effort, learning, and strategic approaches (Dweck, 2006). This belief serves as a key internal asset that can help individuals navigate challenges and pursue long-term goals. The relationship between a growth mindset and future orientation is well documented in the literature. Studies indicate that individuals who endorse a growth mindset are more likely to adopt a long-term perspective and engage actively in goal-oriented behaviors (Rimfeld et al., 2016). Furthermore, they tend to demonstrate stronger stress-management abilities and more effective problem-solving strategies, especially in contexts requiring future planning (Moser et al., 2011). From a motivational perspective, a growth mindset has also been linked to enhanced intrinsic motivation and the use of metacognitive strategies aimed at improving performance (Besty, 2018), which may facilitate sustained efforts toward future objectives.

Adolescents’ development of a growth mindset is influenced by various relational and contextual factors, among which parental autonomy support has been identified as particularly salient. Empirical evidence suggests that parental behaviors such as providing autonomy-enhancing guidance, offering process-oriented feedback, and creating psychologically safe spaces for exploration can strengthen adolescents’ beliefs in the malleability of their competencies (Haimovitz and Dweck, 2016). Warm and autonomy-supportive communication within adaptive family environments may also stimulate metacognitive reflection on personal growth (Zhang, 2022). Neurobiological studies further indicate that consistent parental support can promote functional connectivity in brain regions associated with cognitive control and reward processing (Telzer et al., 2014), which may provide a neural substrate for the development of a growth-oriented belief system.

While the growth mindset framework offers a valuable lens through which to understand how parental autonomy support may foster future orientation, it is important to situate this construct within a broader theoretical landscape. Alternative mechanisms—such as self-efficacy beliefs, goal-setting processes, or systemic interactions across family, peer, and school contexts—may also contribute to the observed relationships. The present study examines growth mindset as one plausible mediator within a larger network of psychosocial resources, acknowledging that its explanatory power may be moderated by individual, contextual, and cultural factors. These findings suggest growth mindset as a potential mediator between parental autonomy support and adolescents’ positive future orientations.

### The mediating role of hope (as an internal asset)

Hope, as another key internal asset for positive youth development, is defined as a cognitive-motivational process for goal attainment (Snyder et al., 2002), involves setting clear goals, generating motivation, and creating strategies to achieve them. As a foundational element for proactive future thinking and goal-oriented actions, hope is associated with positive life attitudes, self-reliance, and psychological well-being (Snyder, 2002). Larson and Luthans (2006) further emphasized that hope facilitates clear goal definition, actionable planning, and persistent effort. Moreover, hope is significantly correlated with career resilience and adaptability (Li and Zhou, 2021), underscoring its role in both personal development and vocational outcomes.

As a malleable psychological construct, hope is influenced by external factors, with parental support emerging as one of the strongest predictors during adolescence (Sui-chu et al., 2021). Adolescents who experience acceptance, appropriate supervision, and autonomy in parent–child relationships, particularly within nurturing and secure family environments, tend to exhibit higher levels of hope (Sulimani-Aidan et al., 2017). Such relational contexts are thought to foster positive internal working models, which support hopeful dispositions. Furthermore, parental autonomy support may enhance future-oriented agency by promoting positive emotional experiences that increase cognitive openness and flexibility in problem-solving (Fredrickson, 2001). Collectively, these findings position hope as a plausible mediator linking parenting behaviors to adolescents’ future orientation. Accordingly, the present study proposes: Hope mediates the relationship between parental autonomy support and adolescents’ positive future orientation.

### The chain mediating role of growth mindset and hope

Empirical research suggests that individuals with a growth mindset are more likely to adopt future-oriented mastery goals and persist in the face of challenges (Peng and Li, 2004; Rimfeld et al., 2016). This cognitive framework encourages sustained effort and constructive responses to setbacks, characteristics that align closely with hopeful agency. Importantly, growth mindset and hope are conceptually distinct: the former pertains to beliefs about the malleability of personal attributes, whereas the latter involves goal-directed motivation and pathway thinking. Yet theoretically, a growth mindset may foster hopeful thinking by encouraging optimistic attributions and proactive coping (Dweck and Yeager, 2019), thereby serving as an antecedent to hope in a developmental sequence. To elucidate the specific mechanism underlying this internal transformation from cognitive belief to motivational resource, we invoke the complementary perspectives of social-cognitive theory, which positions core self-beliefs as primary drivers of action, and expectancy-value theory, which specifies that beliefs about malleability enhance both success expectations and the subjective value of effort, thereby directly fueling the goal-directed agency and pathway thinking central to hope (Bandura, 1997; Eccles and Wigfield, 2002). Building on this integrated perspective, we hypothesize that parental autonomy support may influence adolescents’ future orientation through the sequential mediation of growth mindset and hope. In this study, we examine whether growth mindset and hope operate as sequential mediators between parental autonomy support and future orientation—a hypothesis that extends prior correlational evidence by testing an integrated cognitive-motivational pathway. We acknowledge that this pathway may be moderated by systemic, cultural, or individual factors, and that causal inferences require further longitudinal or experimental investigation.

### The moderating role of peer relationships (as an external asset)

The school environment is a primary microsystem, alongside the family, and serves as an important external asset for early adolescent development. Peer relationships become increasingly salient in early adolescence, as young adolescents begin to shift more of their time and emotional investment toward interactions with peers (Schwartz-Mette et al., 2020). It should be noted that the Developmental Assets Framework has relatively overlooked the important dimension of peer support, whereas previous research and other theories have given considerable attention to the role of the peer environment (Cohen and Wills, 1985). Although peer relationships extend beyond the classroom to include interactions in online spaces, neighborhoods, and extracurricular settings—contexts that may differentially influence motivation and future orientation (Valkenburg and Peter, 2011)—the present study focuses on school-based peer relationships, as school remains the most structured and frequent context for peer interaction among early adolescents in China. In this study, peer relationships refer to the formation of positive interpersonal relationships between individuals of similar age and equal or similar levels of psychological development who collaborate in the process of interpersonal interaction in the classroom system (Zou, 1998).

Empirical research suggests that peer communication plays a crucial role in shaping adolescents’ achievement goal orientation and enhancing cognitive flexibility (Urdan, 1997). While much of the existing literature has focused on negative cognitive patterns—such as findings by Stone et al. (2016), which indicate that strong peer relationships reduce rumination—there has been relatively little attention given to their influence on positive cognitive constructs. Growth mindset, which emphasizes process, development, and intrinsic values over outcome-focused thinking, is a key cognitive and behavioral framework that influences adolescent development. Given the significance of peer relationships in shaping cognitive patterns, their potential role in fostering a growth mindset warrants further exploration.

Hope is a crucial foundation for healthy psychological development in children, with its formation strongly influenced by significant figures in their environment (Scioli et al., 2016). Research indicates that social support from interpersonal networks, including family, peers, and communities, enhances an individual’s sense of hope (Parker et al., 2015). In particular, higher-quality peer attachments are associated with greater levels of hope, as strong peer bonds provide emotional and motivational support. Considering the combined influence of home and school environments, we propose that parental autonomy support has a stronger positive impact on adolescent development when accompanied by high-quality peer relationships. Therefore, peer relationships may serve as a moderating factor in the relationship between parental autonomy support and growth mindset, such that the promoting effects of parental autonomy support on both growth mindset and hope become more pronounced when peer relationships are more supportive.

### Aims and hypotheses

**Figure 1:**
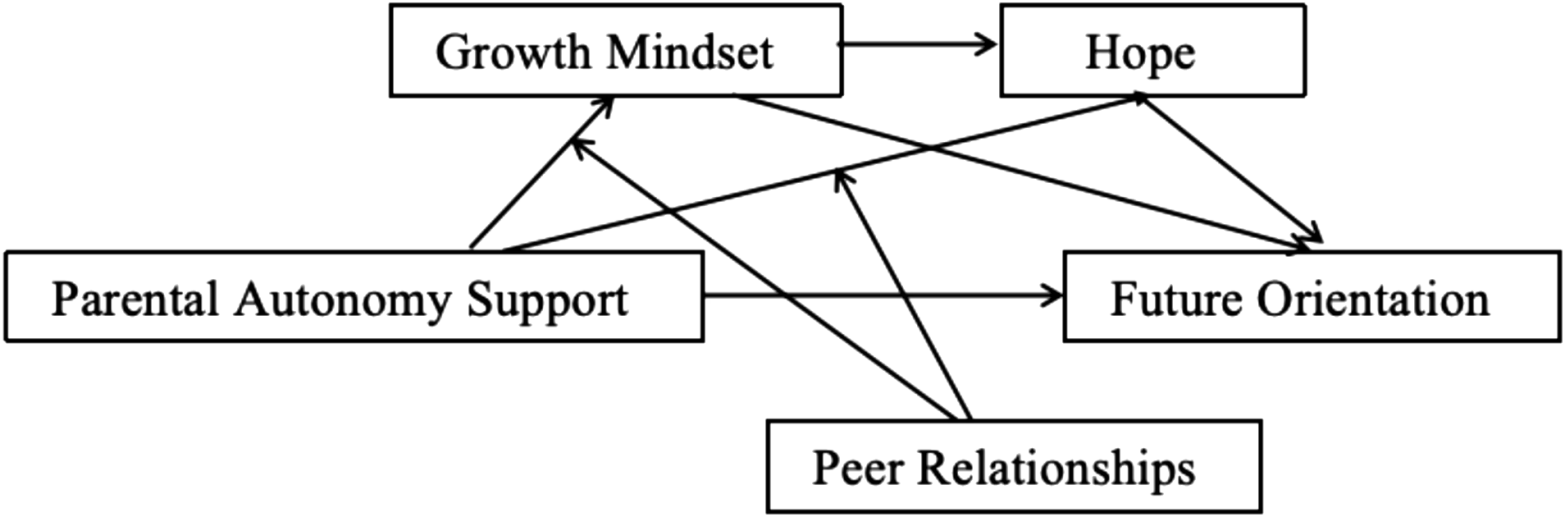
Theoretical model of the current research.

Collectively, this study proposes four research hypotheses and constructs a chain mediation model, as shown in Figure 1:

*Hypothesis 1 (H1)*: Parental autonomy support is directly associated with adolescents’ positive future orientation.

*Hypothesis 2 (H2)*: Parental autonomy support is linked to adolescents’ positive future orientations through the development of a growth mindset.

*Hypothesis 3 (H3)*: Hope mediates the relationship between parental autonomy support and adolescents’ positive future orientation.

*Hypothesis 4 (H4)*: The chain of growth mindset and hope mediates the relationship between parental autonomy support and positive future orientation.

*Hypothesis 5 (H5)*: Peer relationships moderate the relationship between parental autonomy support and growth mindset, such that the association of parental autonomy support on growth mindset and hope is stronger when peer relationships are more supportive.

Based on the positive youth development perspective and the developmental assets framework, this study aims to explore the underlying mechanisms through which parental autonomy support (PAS), as a key external asset, is associated with adolescents’ future orientations (AFO), with a specific focus on the sequential mediating roles of growth mindset and hope—two critical internal assets. By constructing a moderated chain mediation model, the study seeks to deepen the understanding of how external family assets are linked to internal psychological assets in fostering positive development, and to examine how peer relationships, as another form of external asset, moderate these pathways. Furthermore, it provides a scientific basis for family education practices and school-based interventions designed to cultivate adolescents’ positive psychological assets and enhance their future planning capacities. The findings will help inform more balanced strategies between autonomy support and developmental guidance, promote more effective resource allocation, and enhance adolescents’ future readiness. In addition, the study offers empirical support for the improvement of youth development programs and mental health promotion strategies, thereby contributing to the sustainable development of adolescent education.

## Methods

### Participants

This study adopted the questionnaire survey method to survey a total of 625 school students in the first and second grades of three middle schools in the far suburbs of Beijing in the form of anonymous questionnaires. Written informed consent was obtained from the parents or legal guardians of all participants, and assent was obtained from the participants themselves. After excluding invalid data such as regular responses, 604 valid participants (*M*_age_ = 13.3 ± 0.73 years; 303 boys and 301 girls) were finally obtained, with a recovery rate of 96.64%. Among the participants, 242 (40.10%) were urban and 362 (59.90%) were rural. Furthermore, 353 (58.4%) were only children, while 251 (41.6%) were not. To minimize the potential for common method bias and social desirability, several procedural remedies were implemented during data collection (Podsakoff et al., 2003). These included: (1) ensuring participant anonymity and the confidentiality of their responses; (2) clearly stating in the instructions that there were no right or wrong answers; and (3) encouraging participants to respond based on their genuine feelings and experiences.

### Measures

#### Parental Autonomy Support Scale

The Parental Autonomy Support Scale (PASS) revised by Wang et al. (2007) was used. The scale has 12 questions, including three dimensions of providing opportunities for choice, giving explanations, and acknowledging opinions and feelings. The scale is scored on a 5-point Likert scale, with ‘1’ indicating complete non-compliance and ‘5’ indicating complete compliance. Higher scores on the scale indicate a higher level of perceived parental support by the individual. In this study, the Cronbach’s alpha coefficient for the scale was 0.93.

#### Future Orientation Scale

The Future Orientation Scale for Adolescents prepared by Liu et al. (2011) was used, which includes 31 questions divided into three dimensions: future cognition, future emotion, and future volitional action. The scale is scored on a 5-point Likert scale, with ‘1’ indicating complete non-conformity and ‘5’ indicating complete conformity. Higher scores on the scale indicate higher levels of individual future orientation. In this study, the Cronbach’s alpha coefficient for the scale was 0.89.

#### Growth Mindset Scale

The Growth Mindset Scale developed by Dweck (2006) was used, containing six questions, three measuring growth mindset and three measuring fixed mindset. The scale is scored on a 6-point Likert scale, with ‘1’ indicating complete disagreement and ‘6’ indicating complete agreement. Higher scores indicate higher levels of individual growth mindset. In this study, the Cronbach’s alpha coefficient for this scale was 0.75.

#### Hope Scale

The Children’s Hope Scale developed by Snyder et al. (1997) was translated and revised by Zhao and Sun (2011), which consists of six questions and includes two dimensions, namely, motivated thinking and path thinking. It is scored on a 6-point Likert scale, with ‘1’ meaning never and ‘6’ meaning always, with higher scores indicating higher levels of individual hope. In this study, the Cronbach’s alpha coefficient for this scale was 0.91.

#### Peer Relationship Scale

The Peer Relationship Scale (PRS) developed by Zou (1998) was used, which includes the dimensions of peer acceptance, peer fear and low self-esteem. The questionnaire consisted of 30 questions and was scored on a 4-point Likert scale. The first 20 questions were on the peer acceptance subscale, the higher the total score, the better the peer relationship. The last 10 questions are peer fear and low self-esteem subscales with positive scoring, and the higher the total score, the worse the peer relationship. In the present study, the Cronbach’s alpha coefficient for this scale was 0.96.

### Statistical analysis

In this study, SPSS 22.0 was used to perform descriptive statistics and correlation analysis to examine the relationships among parental autonomy support, growth mindset, hope, peer relationships, and future orientation. Common method bias was assessed using Harman’s single-factor test. Subsequently, the SPSS macro PROCESS (Hayes, 2013) was employed to test the hypothesized mediation and moderation effects. Model 6 was used to examine the chain mediating roles of growth mindset and hope, while Model 84 was applied to test the moderated mediation model with peer relationships as a moderator of the paths from parental autonomy support to growth mindset and hope. The bootstrap method with 5,000 resamples was used to test the effects, with significance determined by 95% confidence intervals that did not include zero. In all analyses, demographic variables such as age and family status were included as control variables.

## Results

### Common method bias test

As the data were self-reported from the study participants, a common method bias effect may be introduced. The study used the Harman one-way test to examine this effect. It was found that a total of nine factors had eigenvalues greater than 1, and the maximum factor explained 35.15% of the variance, which is less than 40%, indicating that there is no serious common method bias problem in this study.

### Descriptive statistics and correlation analysis

Correlation analyses showed that parental autonomy support was significantly and positively correlated with hope (*r* = 0.49, *p* < 0.001), growth mindset (*r* = 0.37, *p* < 0.001), Peer relationships (*r* = 0.44, *p* < 0.001), and future orientation (*r* = 0.48, *p* < 0.001); hope was significantly and positively correlated with growth mindset (*r* = 0.37, *p* < 0.001) and future orientation (*r* = 0.59, *p* < 0.001); hope was significantly and positively correlated with growth mindset (*r* = 0.36, *p* < 0.001); and hope was significantly and positively correlated with future orientation (*r* = 0.59, *p* < 0.001); growth mindset was significantly positively correlated with future orientation (*r* = 0.36, *p* < 0.001). Age was significantly positively correlated with parental autonomy support (*r* = 0.12, *p* < 0.001), hope (*r* = 0.12, *p* < 0.001), growth mindset (*r* = 0.08, *p* < 0.001), and future orientation (***r*** = 0.13, *p* < 0.001), and family status was significantly positively correlated with parental autonomy support (*r* = 0.11, *p* < 0.001), and future orientation (*r* = 0.10, *p* < 0.001), and hope (*r* = 0.14, *p* < 0.001), and Peer relationships (*r* = 0.15, *p* < 0.001), were significantly negatively correlated. Age and parental status were used as control variables because they were all correlated with the main variables (Table 1).

**Table 1:** Descriptive statistics and matrix of correlation coefficients for each variable.

Variables	*M*	SD	1	2	3	4	5	6	7	8
1. Gender	–	–								
2. Age	13.3	0.73	−0.06							
3. Situation of only children	–	–	0.19^***^	0.04						
4. Family status	–	–	0.02	−0.01	0.05					
5. Parental autonomy support	3.93	0.88	−0.01	0.12^**^	−0.03	−0.11^**^				
6. Future orientation	3.59	0.56	−0.07	0.13^**^	−0.05	−0.10^*^	0.48^***^			
7. Growth mindset	4.12	1.08	−0.05	0.08^*^	0.01	−0.04	0.37^***^	0.36^***^		
8. Hope	4.50	1.22	−0.08^*^	0.12^**^	−0.01	−0.14^***^	0.49^***^	0.59^***^	0.37^***^	
9. Peer relationships	3.30	0.63	−0.02	0.04	0.01	−0.15^***^	0.44^***^	0.30^***^	0.45^***^	0.48^***^

^*^p < 0. 05, ^**^p < 0. 01, ^***^p < 0. 001, same below.

Gender: male-1, female-0; Situation of only children: only-1, not only-0.

Descriptive statistics, including means, standard deviations, skewness, and kurtosis for all main study variables are presented in Table 2. The absolute values of skewness and kurtosis for all variables were below 1.0, indicating that the data approximated a normal distribution and was suitable for subsequent parametric analyses (Byrne, 2010).

**Table 2:** Descriptive statistics and tests of normality.

Variable	Mean	SD	Skewness		Kurtosis	
			Statistic	Std. error	Statistic	Std. error
Parental autonomy support	3.928	0.880	−0.854	0.099	0.368	0.199
Future orientation	3.591	0.564	−0.496	0.099	0.254	0.199
Hope	4.499	1.219	−0.399	0.099	−0.868	0.199
Growth mindset	4.120	1.081	0.066	0.099	−0.242	0.199
Peer relationships	3.302	0.626	−0.843	0.099	−0.101	0.199

### Analysis of the chain-mediated effects of growth mindset and hope

Linear regression analyses were conducted with age and family status as control variables, parental autonomy support as independent variable and adolescent future orientation as dependent variable. It was found that parental autonomy support was positively associated with adolescents’ future orientation (*β* = 0.47, *t* = 13.06, *p* < 0.001), and hypothesis H1 was valid. On this basis, the regression results of the mediation model are shown in Figure 1. Parental autonomy support positively linked to adolescent future orientation (*β* = 0.23, *t* = 6.03, *p* < 0.001), growth mindset (*β* = 0.37, *t* = 9.63, *p* < 0.001) as well as hope (*β* = 0.40, *t* = 10.63, *p* < 0.001), and growth mindset significantly positively linked to hope (*β* = 0.21, *t* = 5.77, *p* < 0.001), and adolescent future orientation (*β* = 0.11, *t* = 3.24, *p* < 0.001),and hope significantly positively predicted adolescent future orientation (*β* = 0.43, *t* = 11.39, *p* < 0.001).

The results found that the direct effect of parental autonomy support on adolescents’ future orientation was 0. 23, with a 95% confidence interval not including 0 [0. 152, 0. 299], and the three mediating paths of parental autonomy support → growth mindset → future orientation, parental autonomy support → hope → future orientation, and parental autonomy support → growth mindset → hope → future orientation were all significant (The confidence intervals do not contain 0), and the results are shown in Table 3. The total mediation effect was 0.24, the total effect was 0.47, and the effect size of the total mediation effect was 52.01%.

**Table 3:** Analyses of the chain-mediated effects between growth mindset, hope in parental autonomy support and adolescents’ future orientations.

Path	Effect	Effect size	Standard error	95%CI (confidence interval)
Parental autonomy support →Growth mindset →Future orientation	0.04	8.91%	0.01	[0.014, 0.071]
Parental autonomy support →Hope → Future orientation	0.17	35.88%	0.03	[0.124, 0.222]
Parental autonomy support →Growth mindset → Hope → Future orientation	0.03	7.22%	0.01	[0.021, 0.050]

### Analysis of moderated chain mediation effects

After standardizing the variables, PROCESS was used to test the moderating effect of peer relationships, and the results are shown in Table 4. Peer relationships positively predicted growth mindset and the product term of parental autonomy support and peer relationships was significant in predicting growth mindset, suggesting that peer relationships moderated the prediction of growth mindset by parental autonomy support. However, peer relationships, while positively predicting hope, were not significant moderators between parental autonomy support to hope.

**Table 4:** Moderating effects test.

Predictor variable	Growth mindset					Hope				
	*β*	SE	*t*	95%CI		*β*	SE	*t*	95%CI	
				CIL	CIU				CIL	CIU
Age	0.05	0.05	0.99	−0.048	0.144	0.07	0.05	1.55	−0.019	0.159
Family status	0.05	0.07	0.75	−0.846	0.189	−0.14	0.07	−2.11^*^	−0.265	−0.009
Parental autonomy support	0.24	0.04	5.89^***^	0.160	0.320	0.33	0.04	8.43^***^	0.253	0.407
Peer relationships	0.36	0.04	9.11^***^	0.285	0.442	0.12	0.04	3.08^**^	0.043	0.192
Parental autonomy support × Peer relationships	0.09	0.03	2.81^**^	0.028	0.154	0.06	0.03	1.86	−0.003	0.116
*R* ^2^	0.25					0.35				
*F*	39.94					53.33				

For the path “Parental autonomy support → growth mindset → future orientation,” the moderated mediation model determination index INDEX was 0.01, with a 95% confidence interval of [0.002, 0.025], and the moderated mediation effect was significant; For the path “Parental autonomy support → growth mindset → hope → future orientation,” the moderated mediation model determination index INDEX was 0.005 with a 95% confidence interval of [0.001, 0.010], and the moderated mediation effect was significant; For the path “Parental autonomy support → hope → future orientation,” the moderated mediation model determination index INDEX was 0.02 with a 95% confidence interval of [−0.008, 0.062], and the moderated mediation effect was not significant.

The standardized peer relationships were divided into two groups of high and low by plus or minus one standard deviation, and subjects with high and low peer relationships were analyzed separately, and the results are shown in Table 5. For the pathway “Parental Autonomy Support → Growth Mindset → Future Orientation,” the mediating effect was significant in the high peer relationship condition, with 95% confidence intervals of [0.011, 0.071], excluding 0, and the mediating effect was significant in the high peer relationship condition. Again, this was significant in the low peer relationship condition with a 95% confidence interval of [0.004, 0.032]; For the pathway “Parental autonomy support → growth mindset → hope → future orientation,” the mediating effect was also significant in the high peer relationship condition, with 95% confidence intervals of [0.006, 0.030], excluding 0. Again, this was significant in the low companionship condition with a 95% confidence interval of [0.002, 0.015].

**Table 5:** Mediating effects of subjects’ growth mindset at different peer level levels.

Intermediary variable	Moderator variable	Indirect effect value	Boot standard error	Boot CI CIL	Boot CI CIU
Growth mindset	Low peer relationship	0.02	0.01	0.004	0.032
	High peer relationship	0.04	0.02	0.011	0.071
Growth mindset → Hope	Low peer relationship	0.01	0.01	0.002	0.015
	High peer relationship	0.02	0.01	0.006	0.030

**Figure 2:**
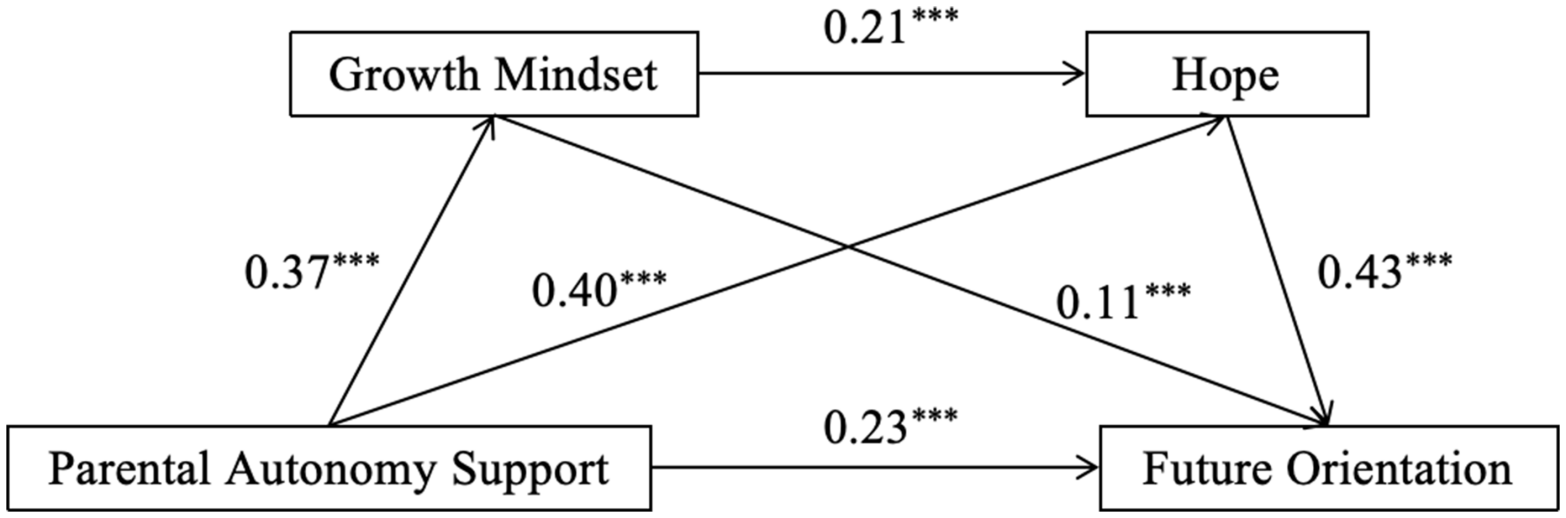
Chain mediation of growth mindset, hope between parental autonomy support and adolescents’ future orientations.

**Figure 3:**
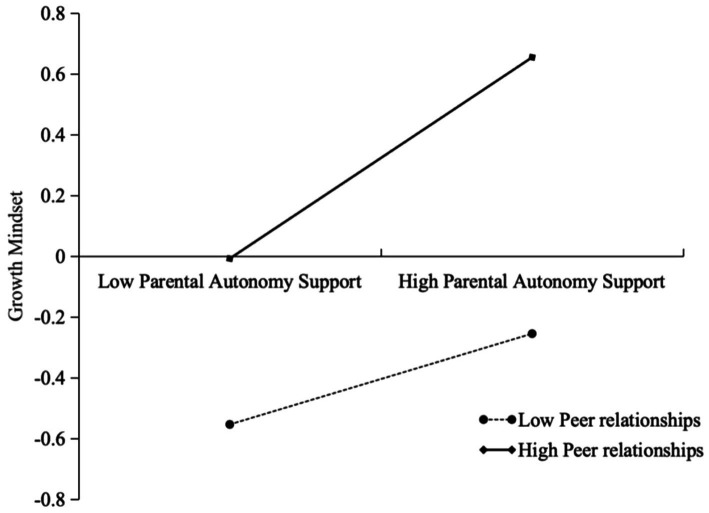
Moderation of the relationship between parental autonomy support and growth mindset by peer relations.

Further simple slope analyses indicated (see Figure 2) that parental autonomy support for high peer relationships was a significant predictor of growth mindset (*simple slope* = 0.33, *t* = 5.79, *p* < 0.001); Similarly, low peer-relationship parental autonomy support was significant in promoting growth mindset (*simple slope* = 0.15, *t* = 3.23, *p* < 0.001) (Figure 3).

## Discussion

Based on the positive youth development (PYD) perspective and the developmental assets framework (DAF), this study developed and empirically tested a moderated serial mediation model. The findings reveal that parental autonomy support, a crucial external asset within the family system, contributes to adolescents’ future orientation not in isolation, but through a sequential psychological process. This process begins with the cultivation of a core belief in the capacity for change, known as a growth mindset. This mindset in turn activates a motivational state of agency and pathways, referred to as hope. Furthermore, the initial stage of this pathway, wherein parental autonomy support fosters a growth mindset, is moderated by the quality of peer relationships, which serves as another key external asset within the school microsystem. Thus, adolescents’ positive development emerges from the synergistic interplay between these two pivotal external assets across family and school systems, providing integrated empirical support for hypotheses H1 through H5.

### Parental autonomy support associating positive future orientation

The current study found that parental autonomy support significantly and positively influences adolescents’ future orientation. This core result provides empirical support for the family ecosystem theory and aligns with prior findings (Umana-Taylor and Guimond, 2010). It demonstrates concretely that when parents engage in child-centered interactions, they help fulfill adolescents’ core psychological needs for autonomy, competence, and relatedness (Deng et al., 2019). This finding clarifies how such a supportive environment operates in practice to nurture self-acceptance, build confidence, and develop essential skills for future planning. According to Self-Determination Theory (Liu and Zhang, 2010), our results illustrate how parental autonomy support acts as a key external asset that can activate adolescents’ intrinsic motivation for growth, thereby strengthening their current well-being and empowering them to face future challenges with a proactive mindset.

Furthermore, this study underscores that when parents actively support autonomy by listening attentively and encouraging independent decision-making, they foster a sense of equality in the parent–child relationship. This observed linkage helps explain how adolescents come to feel valued as individuals, which in turn can strengthen their sense of self-efficacy and build internal psychological resources (Cai and Lian, 2022). From the lens of positive psychology, our findings therefore offer an evidence-based account of how supportive parental interactions enhance adolescents’ psychological capital and self-worth, while potentially reducing anxiety. This nurturing environment, as evidenced by our results, empowers adolescents to face external challenges with resilience, integrate life experiences, and maintain a clear, goal-oriented focus. Overall, our discussion directly connects these observed effects to the cited theoretical frameworks, explaining the mechanism through established constructs rather than presenting theory in isolation.

### The mediating role of growth mindset

This study found that a growth mindset significantly mediates the relationship between parental autonomy support and adolescents’ future orientation. Our results indicate that parental autonomy support positively predicts adolescents’ growth mindset, which in turn is associated with a more positive future orientation. This finding aligns with and extends previous literature by demonstrating that the general climate of autonomy-supportive parenting contributes to a growth-oriented belief system (Hill et al., 2016). We clarify that parental autonomy support here reflects a broad relational style emphasizing respect and encouragement of independence, rather than specific discrete practices such as types of verbal praise. This distinction suggests that the observed effect on growth mindset may be attributable to the overarching supportive climate, consistent with the theoretical emphasis on autonomy need satisfaction as a foundation for adaptive cognitive development.

From the perspective of implicit theories, our mediating finding provides empirical support for the proposition that parenting styles shape adolescents’ core self-beliefs. While experimental research has isolated the impact of specific practices like process-focused praise (Müller and Dweck, 1998), our results highlight the role of a consistent autonomy-supportive environment in fostering the belief that personal qualities can be developed through effort (Dweck, 2006). This belief, in turn, appears to support a more proactive and goal-oriented outlook on the future. However, we acknowledge that this study establishes an associative, not causal, pathway. Alternative mechanisms (e.g., self-efficacy, hope) and contextual moderators (e.g., socioeconomic background) were not tested and may also account for or influence the observed relationships. Future research should examine these possibilities and investigate how growth mindset translates into sustained mastery-oriented behaviors and long-term planning (Molden and Dweck, 2006).

### The mediating role of hope

This study identified hope as a key mediating factor in the relationship between parental autonomy support and adolescents’ positive future orientation. Consistent with earlier research (Hilley et al., 2019; Bi et al., 2022; Snyder et al., 1998), results showed that parental autonomy support was positively associated with hope, which in turn predicted a more optimistic and goal-oriented future orientation. This observed mediation effect can be interpreted through established theoretical lenses. It aligns with the family functioning model (Yang et al., 2016), which posits that security and confidence in harmonious, autonomy-supportive families underlie hopeful thinking, a pattern reflected in the current findings. Similarly, consistent with Fredrickson’s (2001) broaden-and-build theory, the link between autonomy support and future orientation via hope suggests that such parenting may foster an emotional context conducive to cognitive openness and flexibility. Furthermore, the association between autonomy support and hope resonates with Snyder et al.’s (1998) emphasis on relational environments marked by mutual care. In this study, adolescents with stronger hope reported greater belief in their capacity to achieve goals and exhibited heightened resilience and motivation, qualities that were closely tied to a robust future orientation.

### The chain mediating role of growth mindset and hope

This study found that growth mindset and hope function as sequential mediators between parental autonomy support and adolescents’ future orientation, with growth mindset significantly associated with hope. This identified sequential pathway, in which parental autonomy support fosters a growth mindset that in turn cultivates hope, can be interpreted through integrated theoretical perspectives. First, it aligns with the intervention-based framework proposed by Burnette et al. (2022), which positions growth mindset as a foundational cognitive variable that drives motivational outcomes such as hope. Our findings thus provide empirical support for this hypothesized mindset-to-motivation sequence in a naturalistic, non-intervention context. Furthermore, this pathway resonates with broader psychological theories, as it exemplifies Hobfoll et al.’s (2018) concept of resource gain spirals, wherein one key psychological resource facilitates the acquisition of another. This synergistic gain, as noted by Wu (2023), can catalyze further development, and the connection also finds support in neuroscientific understanding. Together, these theoretical insights help explain how the resources nurtured by autonomy-supportive parenting may sequentially build upon one another, ultimately strengthening adolescents’ capacity for future-oriented planning and striving.

### The moderating role of peer relationships

The findings of this study demonstrate that the quality of peer relationships significantly moderates the association between parental autonomy support and growth mindset. Specifically, the positive link between parental autonomy support and growth mindset is stronger among adolescents who report higher-quality peer relationships. This significant moderation effect aligns with Ecological Systems Theory, underscoring how individual development emerges from dynamic interactions across microsystems, including family and peer contexts both within and beyond the school setting, such as in neighborhoods or online social environments. Our results suggest that peer relationships serve as a key contextual amplifier: when peer relational quality is high, autonomy-supportive parenting more effectively fosters a growth mindset, likely through processes of mutual reinforcement and shared valuing of personal development (Franken et al., 2016).

The moderated mediation analysis further revealed that the indirect effect of parental autonomy support on adolescent future orientation through growth mindset depends on peer relational quality. This pathway was evident under conditions of high peer quality but weakened when peer relational quality was low. In contrast, peer relationships did not significantly moderate the association between parental autonomy support and hope. This pattern of selective moderation resonates with the theoretical perspective proposed by Shen et al. (2023), who argue that the synergistic benefits of autonomy and relatedness are most pronounced for adjustment domains closely tied to social-cognitive processes and interpersonal validation. This differential pattern may reflect fundamental differences in the nature and developmental embeddedness of these two mediators. Hope, as a core motivational system, appears more deeply rooted in stable, long-term relational foundations such as early attachment security and internalized patterns of family support (Way and Greene, 2006). During early adolescence, when peer bonds are still forming and may lack emotional depth or consistency, they may be insufficient to modulate a construct as internally anchored as hope. Growth mindset, by contrast, represents a more malleable cognitive framework about ability and effort, one that is particularly responsive to immediate social feedback and validation from peers. Thus, while the influence of parental autonomy support on hope may operate largely through direct internalization, its impact on growth mindset and subsequently on future orientation is more clearly shaped by the quality of adolescents’ current peer relationships.

Furthermore, this study primarily focused on peer relationships as a positive asset. Future research could further explore the complexity of peer influence by, for example, examining whether negative peer relationships (such as peer rejection or deviant peer affiliation) exert a different moderating effect on the association between parental support and adolescent development.

### Implications and limitations

This study has several limitations that warrant attention in future research. First, the cross-sectional design limits the ability to establish causal relationships or capture temporal dynamics among variables. Given that constructs such as growth mindset, hope, and future orientation may fluctuate with changes in family environment and developmental stages, longitudinal tracking or experience sampling methods could be employed in future studies to more accurately depict the evolving mediation patterns over time.t.

Second, the use of self-report measures for all variables may introduce common method bias and fails to distinguish between the distinct roles of fathers and mothers in providing autonomy support. Future research could address these issues by adopting the Actor-Partner Interdependence Model (APIM) and collecting multi-informant data from both parents and adolescents. This approach would help reduce methodological bias while clarifying the individual and interactive effects of each parent, thereby offering a more systematic understanding of family dynamics and adolescent future orientation.

Third, the findings are based on a sample of Chinese adolescents and may be influenced by sociocultural factors specific to this context, such as collectivist values and culturally shaped expressions of parental autonomy support and peer relationships. These cultural characteristics may affect both the meaning and function of the constructs examined, thereby limiting the generalizability of the results to other cultural settings. Future cross-cultural studies are needed to test the universality versus cultural specificity of the observed pathways.

In terms of theoretical implications, by examining the moderating effect of peer relationships as an external asset on parental autonomy support, this study may extend the developmental assets framework, which has traditionally emphasized support from family, adults, and schools, with relatively less attention paid to peer support. These findings help clarify how external developmental resources may contribute to positive developmental outcomes by influencing internal psychological assets, and highlight the potential boundary function of the peer system in the resource transmission process. This research may also deepen the theoretical dialogue between developmental psychology and positive youth development theory, while supplementing the developmental assets framework by illustrating how “external factors operate through internal mechanisms.”

From a practical perspective, the significant mediating roles of growth mindset and hope underscore the potential value of integrating psychological asset cultivation into family education and school support systems. Parent education programs could focus on teaching principles and methods of autonomy support—such as providing choices, offering rationales, and respecting adolescents’ perspectives—to foster positive psychological assets. Moreover, the identified chain mediation pathway suggests that comprehensive intervention strategies addressing both cognitive beliefs and motivational capacities may be particularly conducive to enhancing adolescents’ future orientation. Educational practices could incorporate interventions that promote positive psychological assets, while strengthening collaboration between families and schools to leverage the synergistic effects of parental support and positive peer relationships.

## Conclusion

This study examined the relationship between parental autonomy support and adolescents’ positive future orientation, with a particular focus on the multiple mediation pathways involving growth mindset and hope, as well as the moderating role of peer relationships. The findings offer both theoretical contributions and practical implications, with the main conclusions as follows:

Parental autonomy support demonstrates a significant positive association with adolescents’ positive future orientation. Beyond confirming this fundamental relationship, the study further reveals the important roles of growth mindset and hope in the underlying mechanism: they not only function as independent mediators but also form a complete chain mediation pathway. Additionally, peer relationships show a significant moderating effect between parental autonomy support and growth mindset—in environments characterized by high-quality peer relationships, the association between parental autonomy support and growth mindset is substantially strengthened. However, this moderating effect was not significant in the relationship between parental autonomy support and hope.

Overall, this study enhances our understanding of the connection between parental autonomy support and adolescents’ positive future orientation, and provides a scientific foundation for family education and psychological health interventions targeting adolescents.

## Data Availability

The original contributions presented in the study are included in the article/supplementary material, further inquiries can be directed to the corresponding author/s.
